# Beyond the Sound Waves: A Comprehensive Exploration of the Burn-In Phenomenon in Audio Equipment Across Physiological, Psychological, and Societal Domains

**DOI:** 10.7759/cureus.53097

**Published:** 2024-01-28

**Authors:** Emilian Kalchev

**Affiliations:** 1 Diagnostic Imaging, St. Marina University Hospital, Varna, BGR

**Keywords:** social influences, psychological interpretation, auditory physiology, audio equipment, cognitive biases, sound perception, sound quality, audiophile, audio burn-in

## Abstract

Audio burn-in, often referred to as the process by which audio equipment undergoes a series of played sounds to achieve optimal performance, remains a topic of significant debate within both audiophile communities and relevant scientific fields. While some attribute perceived changes in sound quality to actual physical changes in the equipment, an emerging perspective points to the interplay of physiological, psychological, and social factors that might influence these perceptions. This narrative review delves into the intricate layers of auditory physiology, cognitive sound interpretation, and the wider societal beliefs around burn-in. We underscore the importance of discerning between actual physical changes in audio gear and the multifaceted human factors that potentially modulate our perception of sound. Through a comprehensive exploration, this article illuminates the complexities of this phenomenon, offering insights for both medical professionals and passionate audio enthusiasts and proposing directions for future research.

## Introduction and background

Audio equipment enthusiasts, often self-termed audiophiles, harbor a deep passion for sound - its clarity, depth, texture, and fidelity. Within this community, few topics generate as much discussion, intrigue, and at times contention, as the concept of "burn-in." This term signifies a belief that audio equipment, particularly headphones, in-ear monitors, and speakers, require a "break-in" period during which their sound signature ostensibly matures and reaches its peak performance. Some audiophiles swear by rigorous burn-in protocols, playing certain soundtracks for hours or even days, in the conviction that this will achieve superior auditory output.

However, does the science support this notion? Or are there other underlying factors - physiological, psychological, or social - that might contribute to this perceived enhancement in sound quality? These questions have not only perplexed the audiophile community but have also piqued the interest of professionals in auditory science, cognitive psychology, and even socio-cultural studies [1].

Aims

This narrative review article aims to delve into the rich tapestry of evidence and argument surrounding burn-in. While there is some debate within the audiophile community regarding the physical legitimacy of burn-in - whether mechanical components genuinely undergo changes leading to audible differences - we will primarily center our attention on the human-centric factors. By navigating the intricate pathways of our auditory physiology, the cognitive biases in sound perception, and the socio-cultural influences that shape our beliefs and experiences, we seek to offer a comprehensive understanding of the phenomenon.

Scope and focus

From a medical standpoint, our ears' unique structure and function dictate how we perceive sound. But beyond the mechanics of the ear, the brain plays a pivotal role, interpreting and often altering these perceptions based on prior knowledge, expectations, and beliefs. Furthermore, in a connected world where opinions and reviews are a click away, societal influences, from brand loyalty to peer perceptions, might further modulate our subjective experience of sound [1].

**Figure 1:**
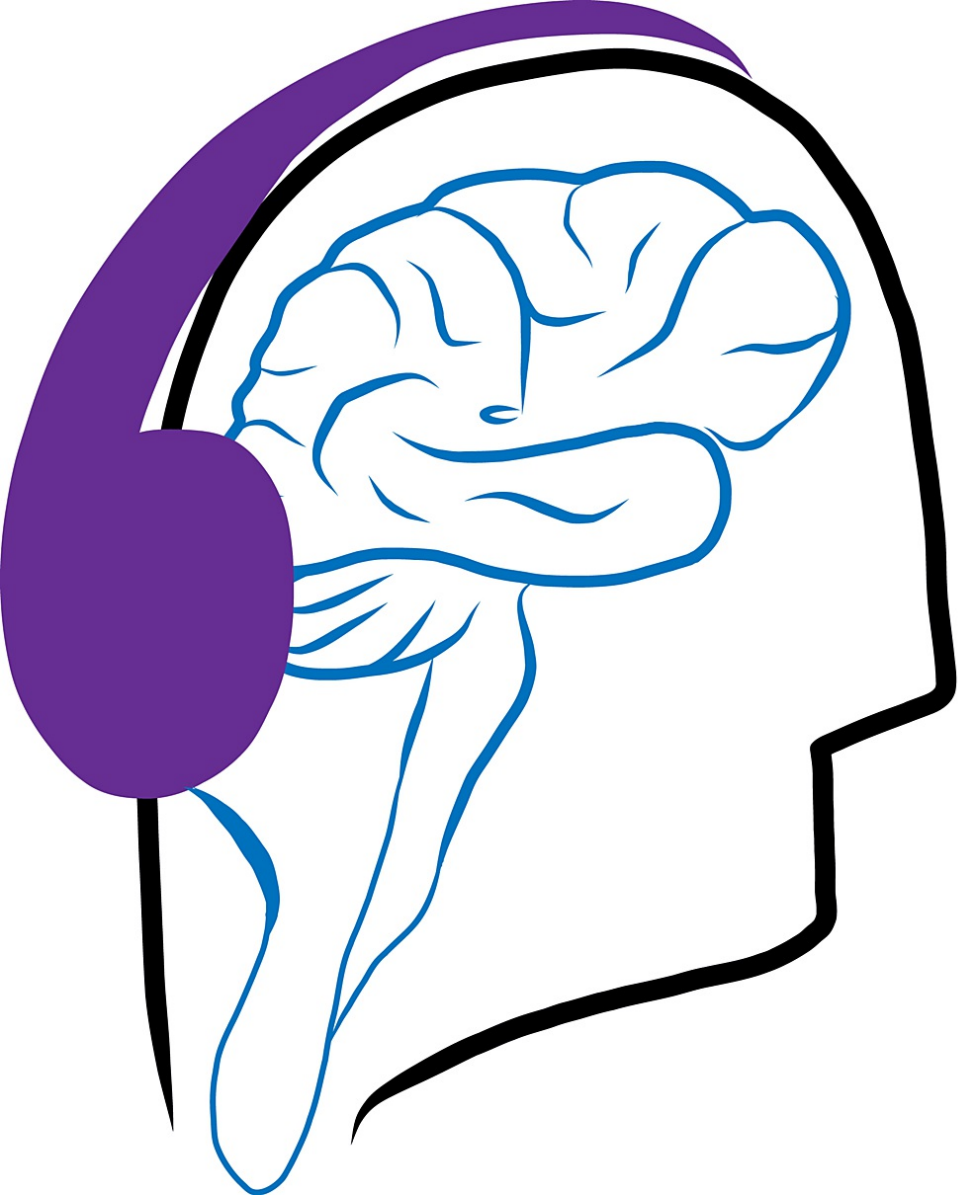
A depiction of the intertwining relationship between auditory stimuli and cognitive processing. Figure created by the author.

By bridging the realms of auditory physiology, cognitive psychology, and social influences, this article seeks to provide clarity on a topic that often sits at the intersection of empirical science and subjective experience. Through this exploration, we aspire not only to inform but to foster a deeper appreciation of the complexities of human sound perception (Figure 1).

In the sections that follow, we will dissect the intricacies of each dimension, aiming to shed light on the multifaceted phenomenon of audio burn-in.

## Review

Physiological factors influencing burn-in perceptions

The Auditory System: Nature's Intricate Design

The development of hearing is a marvel of nature's ingenuity, rooted deep in the ancient tapestry of life. As creatures on Earth navigated diverse ecological niches, auditory systems adapted to meet specific survival needs, culminating in a complex symphony of structures and functions that make up human hearing.

A Historical Look at Hearing

The journey of hearing can be traced back over 500 million years ago to ancient aquatic creatures. Initially, these early vertebrates had simple auditory mechanisms primarily to sense vibrations in their aquatic environments. These vibrations were vital, signaling things like the presence of threats or the location of food sources. As vertebrates transitioned to terrestrial habitats, there was a need for the development of more specialized hearing mechanisms. The challenges posed by aerial sound propagation, with its vast range of frequencies and intensities, required a system that could detect subtle variations in sound [2].

It is posited that the early tetrapods, among the first four-limbed beings on land, had an auditory component resembling the stapes found in modern humans [2]. Over time, some mammals began displaying differences in their auditory systems compared to other creatures [2].

A pivotal moment was the emergence of the three-ossicle chain in the middle ear, enhancing sound amplification and frequency selectivity, especially for high-frequency sounds [3].

These advanced mechanisms allowed some mammals to thrive in environments where acute auditory perception was vital. As generations passed, features like sound localization and fine frequency nuances became essential, especially in places like dense forests where sight might not suffice [3].

Distinctive Features of the Human Auditory System

Humans have further refined many of these evolutionary auditory adaptations. Several features distinguish our hearing capabilities.

Broad frequency range: Humans possess the ability to detect a wide range of frequencies, from as low as 20 Hz to as high as 20,000 Hz. This spectrum, broader than many other mammals, has enriched our acoustic experiences, particularly in complex auditory tasks like speech processing [3].

Mid-high-frequency emphasis: The human ear has heightened sensitivity to frequencies between 2,000 and 5,000 Hz. This range encompasses many of the critical components of speech sounds, underscoring our evolutionary inclination toward effective verbal communication [3].

Sound localization: Our ears are laterally positioned on either side of our head, creating an optimal separation that allows us to pinpoint the origin of sounds with remarkable accuracy. This binaural hearing capability, combined with the sophisticated processing in our auditory cortex, aids in differentiating sound sources in complex environments [4].

Refined cochlear design: The human cochlea, a spiral-shaped organ of the inner ear, houses over 16,000 hair cells, each fine-tuned to detect specific frequencies. This intricate arrangement facilitates our nuanced appreciation of musical melodies and harmonies [3].

The human auditory system stands as a testament to nature's capacity for intricate design. From the deep oceans to the dense forests, hearing has played a pivotal role in the evolutionary saga, with humans inheriting and further refining this legacy for sophisticated auditory experiences.

Anatomy of the Auditory System

The human auditory system, a testament to nature's unparalleled engineering, operates as a finely tuned orchestra, harmonizing the reception, transmission, and interpretation of sound waves. A comprehensive understanding of this intricate structure and its functionality underscores the depth and complexity of auditory perceptions, including phenomena such as burn-in. This journey through the auditory realm initiates at the outer ear, dives into the enigmatic middle ear, and concludes in the labyrinthine depths of the inner ear.

Outer Ear: The Acoustic Antenna

The outer ear, often likened to an acoustic antenna, is our initial point of contact with the myriad sounds that populate our environment. Comprising two main structures - the pinna (or auricle) and the external auditory canal - the outer ear serves as the gateway to our auditory system [5].

Pinna: A cartilaginous, often convoluted structure, the pinna's unique folds and cavities are not merely aesthetic features; they play a pivotal role in sound capture. Its design aids in the following. (1) Directionality: By virtue of its helical shape, the pinna helps in determining the directionality of sounds, especially those originating from above or below [5]. (2) Sound amplification: The concha, the deepest cavity of the pinna, functions to amplify sounds in the 2,000 to 3,000 Hz range, further enhancing our sensitivity to these frequencies [5]. (3) Resonance: The pinna, through its unique contours, aids in creating resonances that amplify specific frequency bands, optimizing sound reception [5].

External auditory canal: Approximately 2.5 cm in length in adults, this tubular passage ends at the tympanic membrane (eardrum). Its primary functions include the following. (1) Sound conduction: It directs sound waves from the external environment to the eardrum [5]. (2) Protection: The canal houses ceruminous glands that produce earwax (cerumen), which protects the ear by trapping debris and has antimicrobial properties [5].

Middle Ear: The Vibrational Nexus

Situated between the outer and inner ears, the middle ear acts as a conduit, channeling sound from the air-filled outer environment to the liquid-filled inner ear. The primary components of the middle ear are the ossicles - the malleus, incus, and stapes [6].

Tympanic membrane (eardrum): Positioned between the outer and middle ear, this thin, semi-transparent membrane vibrates in response to incoming sound waves. Its cone-shaped design, with the apex (umbo) connected to the malleus, ensures optimal transmission of these vibrations to the ossicles [6].

Ossicles: These are the smallest bones in the human body, yet they play an outsized role in sound transmission [6].

Malleus (hammer): Articulating with the eardrum at its umbo, it transmits vibrations to the incus.

Incus (anvil): This bone acts as an intermediary, receiving vibrations from the malleus and conveying them to the stapes.

Stapes (stirrup): The last link in the ossicular chain, the stapes transfers vibrations to the inner ear via the oval window.

Impedance matching: One of the middle ear's most remarkable feats is impedance matching. Given the impedance mismatch between the air-filled external auditory canal and the fluid-filled cochlea, there is a risk of sound energy reflection at this boundary. The ossicular chain and the difference in size between the tympanic membrane and the oval window help in amplifying the force of the vibrations, ensuring efficient energy transmission into the inner ear [6].

Inner Ear: The Sensory Epicenter

Deep within the temporal bone lies the inner ear, an intricate maze of fluid-filled tubes and chambers, chiefly comprising the cochlea and the vestibular system.

Cochlea: Shaped like a snail shell, the cochlea is the auditory portion of the inner ear, responsible for translating mechanical vibrations into electrical impulses [6]. Within its coiled chambers reside the following. (1) Organ of Corti: This sensory structure, nestled on the basilar membrane, houses hair cells, the primary sensory receptors of the auditory system. As the basilar membrane vibrates in response to sound, the hair cells detect these movements and generate electrical impulses [6]. (2) Tonotopic organization of the basilar membrane: The basilar membrane varies in width and stiffness from the base (near the oval window) to the apex (the cochlear tip). This gradient creates a tonotopic map, where different frequencies resonate at distinct locations along the membrane. High frequencies elicit maximal vibrations near the base, while low frequencies peak near the apex [6].

From the outer ear's acoustic funneling to the inner ear's sensory translation, the human auditory system showcases a blend of evolutionary refinement and functional optimization [6]. With such a complex anatomical design, it is plausible that numerous physiological factors might influence our perceptions of sound, potentially impacting phenomena like burn-in [7]. As we journey through this auditory landscape, we are better equipped to understand the physiological intricacies underpinning this enigmatic audiophile experience.

From Sound Waves to Neural Impulses: Biophysics at Play

Sound, in its essence, is a complex interplay of vibrations, transduced and interpreted by our finely tuned auditory system [8]. The journey from ephemeral sound waves to concrete neural impulses involves a fascinating blend of mechanical, bioelectrical, and neural processes. This interplay acts as the foundation upon which our auditory experiences, including perceptions of burn-in, are constructed.

Conversion of Sound Waves Into Mechanical Vibrations

Sound waves, which are essentially variations in air pressure, propagate through the environment until they encounter an obstacle or receptor - in this case, the human ear [8]. As these waves enter the external auditory canal, their energy culminates at the tympanic membrane, setting it into vibratory motion. Depending on the frequency and amplitude of the sound, the eardrum oscillates accordingly [8]. Frequency determines the rate of tympanic membrane vibration. Higher frequencies result in faster oscillations. Amplitude influences the magnitude of displacement or the size of the eardrum's vibrations. These vibrations, in turn, are relayed onto the ossicles, magnifying the sound energy through the mechanical advantage of the ossicular chain, facilitating an efficient transfer to the fluid-filled inner ear [8].

Bioelectrical Processes in Hair Cells: Transduction Galore

Within the cochlear labyrinth lies the organ of Corti, a dynamic assembly of inner and outer hair cells poised to transduce mechanical energy into electrical signals [8]. These cells, crowned with hair-like stereocilia, engage in a dance of bioelectrical wizardry.

Mechanotransduction: As the basilar membrane oscillates due to incoming vibrations, the hair cells' stereocilia sway within the tectorial membrane. This movement causes ion channels located at their tips to open [9].

Influx of positive ions: Predominantly potassium (K^+^) ions rush into the cell. This influx results in a depolarization of the hair cell, creating an electrical potential difference [9].

Release of neurotransmitters: The depolarization triggers calcium (Ca^2+^) channels at the base of the hair cell to open. Calcium's entry into the cell promotes the release of neurotransmitters into the synapse between the hair cell and the nerve endings of the auditory nerve fibers [9].

The Auditory Nerve: Gateway to Cerebral Interpretation

The auditory nerve, or the cochlear nerve, is the first neural conduit in our auditory pathway [3]. Comprising thousands of nerve fibers, each tuned to a specific frequency, this nerve is pivotal in the transduction process.

Synaptic transmission: As neurotransmitters are released from the hair cells, they bind to receptors on the auditory nerve fibers, generating action potentials - the neural language of the brain [3].

Tonotopic organization: Just as the cochlea possesses a spatial mapping of frequencies, the auditory nerve maintains this tonotopic arrangement. Fibers connected to base hair cells (sensitive to high frequencies) and those linked to apex hair cells (sensitive to low frequencies) maintain their spatial segregation as they merge to form the auditory nerve [3].

Transmission to the brain: These action potentials travel along the auditory nerve to the cochlear nuclei in the brainstem, representing the first neural relay station. From here, a series of complex synaptic events will eventually culminate in the auditory cortex, where sound is perceived and interpreted [3].

The journey of sound from external waves to cerebral comprehension is a symphony of biophysical processes, intricately orchestrated. This cascade, from mechanical to bioelectrical to neural, not only allows us to appreciate the world of sound around us but also offers insights into the myriad factors that can modulate our auditory perceptions, including the phenomenon of burn-in.

Adaptation Mechanisms in Auditory Physiology

The human auditory system is an exemplar of both exquisite sensitivity and robustness. It can discern a whisper from a cacophony and adapt to a vast array of acoustic environments. Yet, like any finely tuned instrument, it can experience fatigue and demands periods of recalibration. This adaptation ensures auditory clarity and fidelity, yet, when overwhelmed, might lead to transient or even permanent shifts in auditory sensitivity [6].

Ear Fatigue: The Exhaustion Beneath the Surface

Ear fatigue, often termed "temporary threshold shift" (TTS), is a phenomenon experienced after prolonged exposure to loud sounds [10]. Mechanistically, it is an intricate ballet of cellular and molecular events.

Hair cell overstimulation: Prolonged exposure to elevated sound levels can cause the stereocilia of hair cells to flex excessively. This can lead to ion channel fatigue, resulting in reduced sensitivity to subsequent sounds [10].

Metabolic overdrive: Excessive sound levels demand increased activity from the auditory system's metabolic machinery, depleting energy resources, and leading to transient dysfunction [11].

Disorders Stemming From Maladaptation

The auditory system's delicate balance can be disrupted by chronic or excessive sound exposure, leading to disorders.

Noise-induced hearing loss (NIHL): Persistent exposure to high-decibel sounds can cause permanent damage to the hair cells, particularly when the system's adaptive responses are outstripped. This results in irreversible hearing loss [12].

Tinnitus: Often a sequela of NIHL, tinnitus is the perception of sound, like ringing, without an external stimulus. It is thought to arise due to maladaptive neural plasticity in the auditory pathways [12].

Temporary Threshold Shifts: Recovery and Implications

Temporary threshold shifts are reversible decreases in hearing sensitivity post-noise exposure. Delving deeper:

Mechanism: When overexposed to loud noises, the outer hair cells, pivotal for amplifying soft sounds, temporarily reduce their amplification. This is a protective mechanism, acting like a volume knob turning down, allowing the ear to recover [10].

Recovery: Post-exposure to noise, the auditory system undergoes a "recuperative phase." Depending on the noise intensity and duration, normal sensitivity can return within minutes to several hours. However, repetitive episodes of TTS might reduce recovery efficiency, leading to permanent threshold shifts [13].

Implications for prolonged sound exposure: For audiophiles, understanding TTS is pivotal. Listening to music at high volumes for extended periods might not only induce TTS but also pave the way for NIHL. This has implications for the burn-in phenomenon. If ear fatigue sets in during prolonged listening sessions, perceived changes in sound quality might not wholly be attributed to the audio equipment, but also to the ear's adaptive physiology [14].

In essence, the auditory system's ability to adapt is a double-edged sword. While it protects and recalibrates in the face of varying acoustic environments, excessive demands can lead to maladaptive changes. Understanding this balance is not only vital for preserving auditory health but also offers insights into the nuanced factors shaping perceptions like burn-in [6].

Frequency, Resonance, and the Subtleties of Sound Perception

Sound, at its heart, is a dance of frequencies, each with its distinctive rhythm and cadence. The human auditory system has evolved to discern this vast symphony, from the gentle hum of low frequencies to the piercing sharpness of high pitches [6]. Yet, to understand the intricacies of burn-in from a physiological perspective, one must first delve into the science of frequencies, the phenomenon of resonance, and the cascade of interpretations within our central nervous system.

Frequencies: The Symphony of Sound

Frequencies, measured in Hertz (Hz), reflect the number of sound wave cycles per second. A detailed breakdown is as follows:

Infrasound (below 20 Hz): While these frequencies are below the human range of hearing, they can be felt as vibrations [15]. Typically, they are not the focal point in burn-in discussions.

Low frequencies (20-250 Hz): This range encompasses the bass spectrum, from the deep undertones to the punchier beats commonly found in music [16]. Extended listening sessions, especially at higher volumes, might be most discernible in this range, given the pronounced vibrations they induce [17].

Midrange frequencies (250-2000 Hz): Often considered the "voice" frequencies, this range captures most human vocalizations and many musical instruments [18]. Subtle shifts in perception or nuances in burn-in reports often revolve around this range [19].

High frequencies (2 kHz-20 kHz): The treble spectrum captures the sharpness and clarity of sounds, from cymbal crashes to chirping birds. Age-related or NIHL typically impacts this range first [20].

Resonance: Echoes Within the Ears

Every system, whether architectural or biological, has a natural frequency at which it oscillates most vigorously. This phenomenon, termed resonance [21], is pronounced within the auditory system. The human ear, particularly the ear canal and cochlea, has its resonant frequencies.

Ear canal resonance: Typically lies between 2 kHz and 4 kHz. This amplifies sounds within this frequency range, making them particularly salient [3]. Extended listening might heighten our awareness of subtle changes in this spectrum due to both the equipment's adaptations and our own physiological shifts.

Cochlear resonance: The cochlea does not resonate as a single unit. Instead, its spiral structure and tonotopic organization mean different parts resonate to different frequencies [22]. This intricate design ensures optimal sensitivity across a wide frequency range.

Bridging Physiology and Perception

But sound is not just a mechanical wave, it is an experience [3]. As the frequencies resonate within our ears, they set off a cascade of bioelectrical events, converting these oscillations into neural signals. Yet, the journey does not end in the inner ear. These signals travel up the auditory nerve, passing through key structures in the brainstem, including the cochlear nuclei, superior olivary complex, and inferior colliculi, before reaching the thalamus and finally the auditory cortex in the brain [3]. Along this pathway, the signals are processed at various stages, integrating with descending pathways and other sensory inputs. In the auditory cortex, they are interpreted and compared to past memories, forming our complex auditory experience.

While this section delves deep into the mechanics of sound perception, the subsequent sections will unveil the cognitive artistry behind it. Our brains do not just process sound, they paint an auditory landscape, replete with emotions, memories, and anticipations [23]. This complex interplay between ear and brain, physics and perception, forms the crux of our understanding of phenomena like burn-in.

Frequencies and resonance are foundational to our sound experiences. Yet, they are just one piece of the puzzle. As we journey from the external world to the realms of cognition, the layered intricacies of burn-in will further unravel.

Physiological Perspectives on Burn-In: Synthesizing Knowledge

Burn-in, as a concept, has long intrigued and perplexed the audiophile community. While the debate over mechanical changes in audio equipment endures, the real crux of the matter may lie within the listeners themselves. Delving deep into our auditory system’s physiology provides insights, both evident and subtle, about how our bodies and brains might experience and interpret prolonged sound exposure.

Prolonged Exposure: A Dance of Adaptation

Firstly, consider the potential repercussions of extended audio playback. Chronic sound exposure, especially at high volumes, can induce ear fatigue, with hair cells in the cochlea experiencing stress [24]. Over time, this could lead to temporary threshold shifts. While these shifts usually revert, they raise an intriguing question: Could this transient alteration in sensitivity be misconstrued as a change in the audio equipment's sound profile? Essentially, rather than the device "burning in," it is conceivable that our auditory system temporarily "burns out," reducing its sensitivity and thereby altering sound perception [24].

Moreover, it is paramount to appreciate that the ear is not just a passive recipient of sound. It actively fine-tunes our auditory experiences through mechanisms like the efferent system, which dampens hair cell activity based on environmental sound levels [25]. Extended listening might engage this system more robustly, refining our perception over time.

Auditory Variability: Unique Ears, Unique Experiences

The concept of individual variability is a cornerstone in medical science, and the auditory system is no exception. Our ears, though functionally consistent across humans, have nuanced anatomical differences/variations in ear canal length and shape, slight differences in the ossicular chain, or even subtle diversities in cochlear innervation [26]. All these factors culminate in a unique auditory signature for each individual.

It is plausible that this individualized anatomy and physiology, when exposed to the same audio stimuli, would react slightly differently. Some may experience more pronounced ear fatigue, others might have heightened sensitivity to certain frequencies due to their unique resonance patterns [26]. Consequently, two individuals, using the same headphones for the same duration, might report distinct burn-in experiences.

Beyond Mechanics: The Human Element of Burn-In

In light of the lack of substantial evidence supporting mechanical changes during burn-in, the onus shifts to human physiology. Our auditory system is a marvel of evolutionary engineering, designed not just for sound reception, but for optimization and adaptation [3]. While it is tempting to attribute perceived sound changes solely to the equipment, the labyrinthine complexity of our ears and the dynamic processes within them suggest a more nuanced narrative. The burn-in phenomenon, when viewed through the lens of human physiology, becomes a symphony of potential adaptive mechanisms, individual variabilities, and intricate biophysics [27].

Psychological dimensions of burn-in

Neural Underpinnings of Auditory Perception

The auditory system's complexity extends far beyond the intricacies of the ear, delving into a vast neural network woven to decode the myriad sounds our environment produces [6]. As sound waves are captured and transformed into electrical signals by the inner ear, a series of neurological events ensue, leading to our conscious perception of sound.

Auditory Pathways: From Ear to Corte

Post the inner ear's initial processing, the spiral ganglion neurons receive these electrical impulses, transmitting them to the brainstem via the auditory nerve. The first port of call is the cochlear nuclei, a paired structure located at the juncture of the pons and medulla oblongata. It is here that the initial segregation of auditory information begins, preparing it for its journey through higher neural centers [6].

From the cochlear nuclei, sound signals diverge along various pathways. One critical relay point is the superior olivary complex. This nucleus plays a pivotal role in localizing sound in space, particularly in deciphering the time differences in sound reaching both ears [6].

The auditory signal’s voyage continues upward to the inferior colliculus in the midbrain. Not only does this nucleus act as a waypoint but also integrates auditory information with sensory input from other modalities, providing a more holistic sense of our surroundings [6].

**Figure 2:**
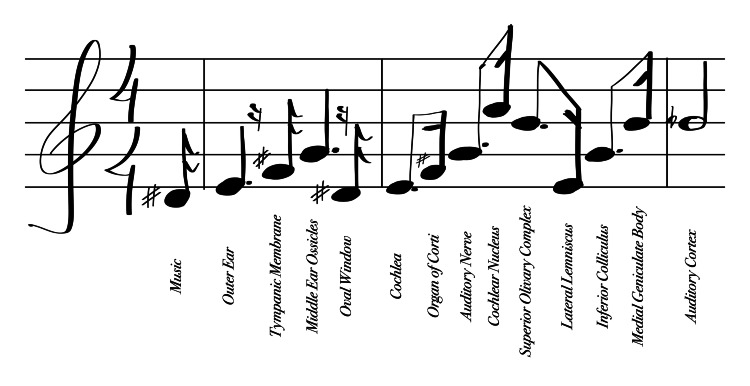
An illustrative journey through the auditory pathway. This diagram creatively uses a sequence of musical notes to represent the auditory processing pathway, with each note corresponding to a specific step in the process – from "Music" through to the "Auditory Cortex." This depiction aims to visually and conceptually bridge the art of music with the science of auditory perception. Figure created by the author.

As we ascend the auditory hierarchy, the medial geniculate body of the thalamus acts as a final relay before the cerebral cortex. This structure filters and directs the signals to the appropriate regions of the auditory cortex, ensuring efficient processing (Figure 2) [6].

Frequency and Intensity: Cortical Representations

Upon reaching the auditory cortex - nestled within the Sylvian fissure on the superior temporal gyrus - the true intricacies of sound perception become evident. Here, neurons are organized in a tonotopic manner, meaning they are spatially arranged based on the frequency they respond to. Lower frequencies are processed anteriorly, and higher ones posteriorly. This specific organization ensures that we can discern between various pitches, from the deep notes of a bass guitar to the high trills of a piccolo [28].

Alongside frequency, intensity or loudness is another crucial component of sound. Neurons in the auditory cortex respond to specific sound intensities, with some attuned to softer sounds and others to louder ones. This gradation ensures we can discern a whisper from a shout, allowing for nuanced auditory experiences [28].

Auditory Plasticity: The Ever-Adapting System

The auditory system is not a static entity; it exhibits remarkable plasticity. Neural plasticity refers to the brain's ability to reorganize itself by forming new neural connections. This trait is evident in the auditory system as our experiences continually shape it [29].

For instance, musicians often show enhanced auditory cortices, with specific areas enlarged due to repeated exposure and focus on certain sound frequencies. Such changes underline how our experiences can remodel our neural architecture, preparing us for similar future stimuli [29].

The idea of plasticity is pivotal when considering burn-in from a psychological standpoint. If the auditory system can adapt based on experience, it is plausible that repeated exposure to a particular sound signature might induce neural changes, thereby altering subsequent sound perceptions [30].

In essence, our understanding of auditory perception is rooted in the harmonious collaboration between the ear and the brain. The intricate neural tapestry not only decodes sounds but also continuously adapts to them, ensuring that our auditory experiences remain dynamic and ever-evolving.

Memory, Experience, and Sound Perception

The phenomenon of listening extends beyond mere sound wave detection and conversion into neural impulses. The intricacy of auditory perception is underscored by the profound influence of memory and prior experiences on how we perceive and interpret sounds [31]. This interaction, rooted in the neural dynamics of the brain, offers insights into the subjective nuances of auditory experiences, which can be particularly pertinent when considering phenomena such as burn-in.

Auditory Cortex and the Tapestry of Sound Memories

Situated within the Sylvian fissure on the superior temporal gyrus, the auditory cortex does not just passively process incoming sound signals [32], it actively engages in recognizing and recalling previously heard sounds. Whether it is a familiar melody, the cadence of a loved one's voice, or the distinctive chirp of a specific bird, the auditory cortex plays an indispensable role in recognizing these sounds based on stored memory patterns. Through repeated exposures, neural networks within this region get attuned to specific sound signatures, facilitating quicker and more nuanced sound recognition in subsequent encounters [32].

Prior Auditory Experiences: Coloring Sound Perceptions

Every auditory experience, be it mundane or profound, leaves an imprint on our neural architecture. These imprints, or memory traces, shape subsequent sound perceptions. For instance, the way one perceives the tonal quality of a new audio device might be profoundly influenced by the sound signature of their previous device. This effect stems from the brain's inherent tendency to compare incoming sensory information with stored memory patterns, seeking familiarity and patterns [33].

In the context of burn-in, if an individual has strong prior experiences or beliefs about how a device "should" sound after a burn-in period, they are more likely to perceive subtle differences, even if objectively, no significant change has occurred [34]. It is a dance of expectation, recollection, and perception, choreographed intricately by the neural circuits.

Hippocampus and Auditory Regions: Crafting Sound Memories

While the auditory cortex is the primary hub for sound processing and recognition, it does not work in isolation. The hippocampus, a structure deeply embedded within the brain's temporal lobe and a key part of the limbic system, plays a cardinal role in memory formation and retrieval [35]. This region, reminiscent of a seahorse in its shape, collaborates closely with the auditory cortex when it comes to sound-based memories.

Listening to a song can suddenly evoke vivid memories of a past event. This phenomenon, known as "evocative memory," results from the hippocampus retrieving associated memories triggered by the sound. The auditory cortex provides the hippocampus with the sound's neural signature, and the hippocampus responds by surfacing related experiences stored within its intricate neural networks [35].

Furthermore, the act of consciously recalling a specific sound or melody, say, for instance, trying to remember how a particular song goes, involves a neural dialogue between the hippocampus and the auditory cortex [35]. It is a testament to the seamless integration of memory and auditory systems, working hand in hand to give depth and context to our auditory experiences.

Sound perception is not a mere passive reception of auditory signals. It is an active, dynamic process shaped by memories, prior experiences, and intricate neural dialogues between the auditory and memory systems of the brain. Understanding this interplay offers a richer perspective on why individuals might have varied and deeply subjective auditory experiences, including perceptions related to burn-in.

Cognitive Biases in Auditory Perception

At the heart of understanding any human perception, including the auditory domain, lies the intricate dance between raw sensory input and the cognitive processes that shape and interpret it [31]. Within this framework, expectation, prior knowledge, and cognitive biases emerge as potent modulators of how we experience sound. These influences become especially pronounced in contexts like burn-in, where the intersection of objective auditory experiences and subjective cognitive processes becomes overtly evident.

Expectation and Prior Knowledge: Setting the Stage

Before delving into specific biases, it is essential to recognize the overarching influence of expectation and prior knowledge. The brain, being an anticipatory organ, constantly makes predictions about the external world [36]. In the auditory realm, if one expects a newly purchased audio device to sound better after a certain period, this very expectation might prime the auditory cortex to perceive sounds more favorably. Such anticipatory neural dynamics are rooted in the brain's desire to align sensory input with prior knowledge and beliefs, ensuring cognitive coherence [36].

The Placebo Effect: Belief as a Powerful Modulator

One of the most researched cognitive biases in medicine and psychology, the placebo effect, holds relevance in the auditory domain as well [37]. In essence, the placebo effect emerges when belief in a treatment's efficacy (even if the treatment is inert) leads to tangible physiological changes or perceived benefits.

Translating this to the realm of burn-in, if an audiophile believes that their headphones will attain optimal sound quality after, say, 50 hours of play, this very belief might induce genuine perceptual changes in how they experience the sound, even if there is no measurable alteration in the device's output. The placebo effect underscores the brain's capacity to modify sensory experiences based on belief alone [37].

Confirmation Bias: Hearing What We Want to Hear

Another cognitive bias, the confirmation bias, predisposes individuals to seek, interpret, and recall information in ways that affirm their pre-existing beliefs [38]. In the context of burn-in, once an audiophile expects or believes in a change in sound quality post the burn-in period, they are more likely to notice sound attributes that confirm this belief, while potentially overlooking details that might refute it. This selective attention and interpretation further entrenches the belief in burn-in, even in the absence of objective evidence [38].

"Brain Burn-In": Cognitive Acclimatization Over Time

While the aforementioned biases often stem from external influences and personal beliefs, there is an intrinsic cognitive phenomenon that also plays into the burn-in narrative: the so-called "brain burn-in.” This concept posits that our brains, through repeated exposure to a specific sound signature, undergo a form of neural acclimatization [39].

For instance, if an individual listens to a new audio device continually, the initial novelty or unfamiliarity of its sound profile might diminish over time, not necessarily due to any change in the device itself but because of the brain's adaptive recalibration. Such repeated exposures might subtly shift auditory expectations and perceptual baselines, making the device sound more "familiar" or "natural" over time, even if its acoustic output remains constant [39].

When exploring the complex terrains of burn-in, it becomes imperative to understand that what we hear is not just a result of external acoustic signals but is also deeply influenced by our cognitive landscapes. Biases, expectations, and neural adaptations interplay in multifaceted ways, creating a rich tapestry of auditory experiences, some of which can be easily misattributed to external factors like burn-in. Recognizing and dissecting these cognitive dimensions offers a holistic and nuanced understanding of the burn-in phenomenon.

Emotion, Mood, and Sound Interpretation

Music and sound have long been known to elicit profound emotional reactions in humans, from the haunting melodies that invoke nostalgia to the upbeat rhythms that compel us to dance [40]. The intertwined relationship between auditory perception and emotions offers an intricate landscape for exploration, especially in the context of burn-in where prolonged listening experiences might intersect with various emotional states. This synthesis of sound and emotion is not a mere coincidence but is deeply rooted in our neurobiological architecture.

The Limbic System: Orchestrating Emotional Responses

At the nexus of sound and emotion stands the limbic system, a collection of intertwined brain structures responsible for generating and regulating emotions. Two primary components within this system, i.e., the amygdala and the hippocampus, have been particularly linked to auditory-emotional processing. When a sound, whether it is a song or a specific frequency, enters our auditory pathway, it does not just remain a mere sensory stimulus. The amygdala assesses its emotional significance, generating feelings of pleasure, discomfort, or even neutrality [41].

Further, the hippocampus, with its role in memory, often associates sounds with past events or experiences [42]. A particular song or sound frequency might transport a listener back to a past moment, invoking the emotions felt at that time. This emotional memory integration further deepens the experience of sound, moving it beyond mere sensory perception.

Bi-directional Influences: Emotions as Sound Modulators

The relationship between sound and emotion is dynamic and bi-directional. Just as certain sounds can invoke specific emotional states, our pre-existing moods can also influence how we perceive sound. Someone in a melancholic mood might interpret a song's lyrics more profoundly, perceiving nuances that they might overlook when in a happier state. This phenomenon can be partially attributed to the brain's salience network, which, influenced by our emotional state, determines the significance or relevance of external stimuli, including sound [43].

Moreover, emotions, being states of heightened physiological arousal, can subtly alter our auditory sensitivity. For instance, increased stress levels (and the associated cortisol release) can sharpen our auditory acuity, potentially making us more sensitive to specific frequencies or nuances during listening [44].

Implications for Burn-In: Emotion's Pervasive Influence

The complex interplay between auditory perception and emotion holds implications for the burn-in discourse. When audiophiles engage in prolonged listening sessions, especially during the so-called burn-in periods, their emotional states can fluctuate. A relaxed, contented listener might perceive the sound differently from their stressed or agitated counterpart, even if the audio equipment remains unchanged [43].

Furthermore, the very act of investing in new audio equipment might come with an emotional charge - excitement, anticipation, or even skepticism. These emotions, whether they stem from the expectation of enhanced sound quality post burn-in or from other sources, can act as filters, subtly modifying the auditory experience.

If, for instance, an individual feels immense satisfaction from their new purchase, this positive emotional state might amplify the perceived benefits of burn-in, even if the actual acoustic differences are minimal or non-existent. Conversely, a listener skeptical about burn-in might unconsciously seek flaws or remain indifferent to potential enhancements in sound quality [45].

The sounds we hear are not mere frequencies striking our eardrums but are deeply imbued with emotional significance. Whether it is the limbic system's integrative processing, the influence of past memories, or the modulation of perception by our current mood, our auditory experiences are rich, multi-dimensional tapestries woven with threads of emotion [43]. In understanding burn-in and its potential effects, we must not only focus on the hardware or the cognitive biases but also appreciate the profound emotional landscapes that each listener brings to the auditory table. Recognizing this intricate dance between sound and emotion offers a more holistic, nuanced perspective on the burn-in phenomenon.

Synthesis: A Psychological Perspective on Burn-In

The realm of auditory perception is a rich tapestry woven with threads of both physiology and psychology. The burn-in phenomenon, commonly discussed within audiophile circles, serves as a fitting lens through which we can examine the intricate dance of these intertwined domains [30]. As we seek to unravel the mystery of burn-in, it becomes apparent that our sound experiences are not merely the product of vibrating air molecules entering our ears, but are profoundly shaped by the labyrinthine corridors of our minds.

The Psychological Symphony of Burn-In

At the core of our understanding lies the realization that the brain is an active interpreter, not a passive receptor [46]. While the ear captures sound waves, it is the brain that paints the auditory picture, and this process is deeply influenced by an array of psychological mechanisms. From the neural underpinnings that trace the auditory pathways beyond the ear to the critical roles of memory and experience in shaping sound perceptions, the brain is actively molding our auditory experiences every step of the way [32].

Couple this with the cognitive biases that we, often unknowingly, carry with us. The placebo effect, where mere belief in improvement can lead to perceived enhancement, and confirmation bias, where we seek out evidence that aligns with our beliefs, can significantly skew our auditory experiences. The phenomenon of "brain burn-in," a recalibration of our auditory expectations through repeated exposure, further underscores how malleable our sound perceptions truly are [38].

Intertwining Physiology With Psychology

But this psychological symphony does not play in isolation. It is harmoniously intertwined with the very physiology of our auditory system. The nuances of frequency perception and the subtleties introduced by resonance phenomena have profound implications on how we perceive changes in sound quality [47]. Meanwhile, our emotional states, governed by the deep recesses of our limbic system, can further modulate this perception, adding another layer of complexity [43].

This duality becomes especially pertinent when considering adaptation. At the physiological level, auditory adaptation refers to the ear's response to prolonged sound exposure, potentially leading to phenomena like ear fatigue [48]. Contrastingly, at the psychological level, adaptation encompasses the brain's ever-evolving interpretation of sound, influenced by factors ranging from expectation to previous auditory experiences. While the former might result in temporary threshold shifts, the latter can redefine the very auditory landscapes we traverse [8].

Drawing the Line and Seeing the Overlap

As we stand at the crossroads of physiology and psychology, it becomes essential to distinguish between these realms while also acknowledging their overlap. The burn-in experiences narrated by audiophiles worldwide might not always find grounding in the mechanical changes within audio equipment. Still, they do find resonance in the adaptable, intricate nature of human auditory perception [45]. Whether these changes stem from the ear's adaptation mechanisms or the brain's interpretative dance, they remain valid facets of the listening experience.

To truly grasp the essence of burn-in, we must not merely tune into the sounds around us but also attune ourselves to the symphony playing within, where psychology and physiology converge in harmony.

Societal and community perspectives on burn-in

The Social Neuroscience of Group Behavior and Beliefs

The human brain, renowned for its complexity and adaptability, is wired not just for survival but for social connection. Social neuroscience, an interdisciplinary field at the nexus of social psychology and neuroscience, provides valuable insights into how social behaviors and cognition are underpinned by neural mechanisms [49].

Neural Basis of Social Interactions and Group Behavior

From an evolutionary standpoint, the ability to cooperate and function as a cohesive unit has always conferred a survival advantage [50]. It is not surprising, then, that the brain has distinct regions dedicated to understanding, interpreting, and reacting to social stimuli. The temporoparietal junction (TPJ), anterior cingulate cortex (ACC), and orbitofrontal cortex (OFC) are a few regions that have been consistently implicated in social cognition [51]. For example, the TPJ plays a critical role in perspective-taking and theory of mind [52] - the ability to attribute mental states to others. The ACC, on the other hand, is more attuned to evaluating social errors, like when we recognize we have breached a social norm [53].

In addition to these roles, the TPJ's involvement in perspective-taking is crucial for empathizing with others, while the ACC's role in evaluating social errors helps in navigating complex social dynamics. Neuroimaging studies consistently show heightened activity in these areas when individuals process social information, be it understanding societal norms, empathizing with others, or even conforming to group behaviors [54]. Such neurobiological architecture points to our inherent need to belong and, often, align our beliefs with those of a larger group.

Collective Beliefs and Shared Narratives: Influencing Individual Cognition

Communities, whether they revolve around hobbies, professions, or shared experiences, offer collective narratives that influence individual cognition profoundly. Consider the "echo chamber" effect seen on many online platforms, where similar ideas get reinforced while dissenting voices are drowned out [55]. Within such environments, individuals are more likely to encounter reinforcing stimuli, leading to a strengthening of particular neural pathways associated with these beliefs. The phenomenon is reminiscent of Hebbian theory - neurons that fire together wire together [56].

Moreover, the neurochemical oxytocin, often dubbed the "social hormone," has been shown to play a pivotal role in trust and group bonding [57]. Elevated levels of oxytocin can enhance feelings of trustworthiness toward in-group members while promoting skepticism toward those outside the group [58]. In the context of audiophile communities or any tight-knit group discussing burn-in, the increased trust in peer experiences and shared beliefs, potentially mediated by oxytocin and other neurochemicals, may amplify adherence to certain narratives, including those about burn-in.

Such neurobiological insights into group behaviors and shared beliefs make it clear: our perceptions and beliefs, even in domains as seemingly objective as auditory experiences, are not formed in isolation. They are continually shaped, molded, and reaffirmed by the neural interplay of personal experiences and collective narratives. As we delve deeper into the sociocultural aspects of burn-in in the sections that follow, it is imperative to keep in mind this neural backdrop, for it underscores the intricate dance of individual cognition within the broader ballroom of societal influences.

Audiophile Communities as Social Constructs

The human propensity for forming communities around shared interests has been a foundational cornerstone of societal structures [59]. One such community that has garnered both enthusiasm and skepticism in equal measure is the audiophile community. At its heart, this community is united by a profound passion for sound quality, but like any other social construct, it is subject to the influences of shared narratives, influential members, and collective memories.

Formation and Evolution of Audiophile Communities

Historically, communities formed around geographic proximity, but with technological advancements, particularly the internet, virtual communities based on shared interests have flourished [60]. Audiophile communities trace their roots back to the analog era - the golden age of vinyl and high-fidelity sound systems [61]. With the advent of the digital age and online forums, these communities have expanded, diversified, and found new platforms for interaction.

These online platforms, ranging from discussion boards to dedicated websites and social media groups, serve as hubs where audiophiles share experiences, discuss equipment, and seek advice. Over time, certain norms, terminologies, and beliefs take root, with "burn-in" being one such widely discussed phenomenon [62].

The Power of Shared Experiences: Collective Validation and Communal Memory

Within the audiophile community, personal experiences with audio equipment become more than isolated events. When shared, they contribute to a communal memory, a collective repository of experiences and knowledge [63]. As members share their burn-in experiences, patterns emerge. For a newcomer, this vast pool of shared knowledge offers a sense of validation. If numerous community members attest to changes in sound quality over time, it offers a form of collective validation, reinforcing the burn-in narrative.

But it is not just the sheer number of similar experiences that reinforce beliefs, it is the emotional resonance of these shared narratives. Stories of dramatic transformations in sound quality or long waiting periods for optimal performance can evoke strong emotional responses, further ingraining the burn-in phenomenon into the community's collective consciousness [64].

Impact of Influential Members or "Gatekeepers" on Community Beliefs

Within any community, certain members wield more influence than others [65]. These influential members, sometimes referred to as "gatekeepers," can be veteran audiophiles, professional reviewers, or individuals with a significant online following. Their opinions, reviews, and experiences can substantially shape community beliefs and norms.

If a gatekeeper shares a positive burn-in experience, it might be perceived as more credible or valuable than several accounts from less influential members. Their endorsement can lend significant weight to the burn-in narrative. Conversely, skepticism from a gatekeeper can spark debates and challenge established beliefs.

However, it is crucial to note that these influential figures, while impactful, are still part of the larger social construct. Their beliefs and experiences are also shaped by their interactions within the community and their personal biases.

In summary, audiophile communities, as with any other interest-based community, are complex social constructs. While united by a shared passion, they are influenced by collective narratives, the emotional resonance of shared experiences, and the opinions of their influential members. Understanding these dynamics offers a nuanced perspective on why certain beliefs, like the burn-in phenomenon, gain traction and become deeply entrenched within the community.

Peer Influence and Cognitive Conformity in Burn-In Beliefs

Understanding human behavior often requires a multidisciplinary lens, melding perspectives from neuroscience, sociology, and psychology. Peer influence, a phenomenon central to shaping beliefs within communities, stands as a testament to this intricacy [66]. Audiophile communities, in discussing topics like burn-in, are no exception. Peer influence within such communities can drive cognitive conformity, leading individuals to align their perceptions and beliefs with the prevailing majority.

Social Psychology's Perspective on Peer Validation

Social psychology, a branch deeply rooted in understanding interpersonal relationships and social behavior, offers insights into how individuals navigate the waters of peer influence. Humans, as social beings, possess an intrinsic need to belong [67]. This drive often manifests as seeking validation from peers, especially within interest-based communities.

Within audiophile communities, this validation can stem from shared experiences. When a member's experience or belief about burn-in aligns with the majority, it reinforces their stance. Conversely, when one's belief deviates, the community might exert subtle or overt pressures to "correct" this deviation. This dynamic is not unique to audiophiles but rather a universal trait across various communities.

Cognitive Conformity: The Alignment Mechanism

Cognitive conformity is a phenomenon where individuals adjust their beliefs or perceptions to match those of a group. Several mechanisms underpin this behavior.

Informational influence: This arises from a genuine belief that the group possesses more accurate information. In the context of burn-in, if the majority attest to its validity, a newcomer might believe this collective experience outweighs their initial skepticism [68].

Normative influence: Here, the individual conforms not because they believe the group is correct but because they seek social approval or fear social rejection [69]. An audiophile might publicly endorse burn-in beliefs to fit in, even if privately skeptical.

Reference groups: These are groups that individuals aspire to join or identify with [70]. If an aspirational group within the audiophile community staunchly believes in burn-in, it might influence members to adopt similar beliefs.

Peer influence and cognitive conformity are powerful drivers in shaping individual beliefs within audiophile communities. The intricate dance between personal experiences, the need for validation, and community pressures crafts a dynamic landscape, explaining the fervor and debate surrounding burn-in. By understanding these social and cognitive underpinnings, one gains a holistic perspective on why and how burn-in beliefs persist and evolve.

Marketing, Branding, and the Power of Suggestion

In our exploration of societal and community influences on burn-in, we must address the potent role of marketing and branding. Within the complex web of factors molding our auditory perceptions and beliefs, the power of suggestion through strategic marketing campaigns is profound. Not only does it influence purchase decisions, but it also crafts narratives that shape our expectations and experiences of the products we use [71].

Psychology of Marketing: A Primer

The psychology underpinning marketing is intricate [72]. Marketers tap into a myriad of cognitive and emotional triggers to drive consumer behavior. In the realm of high-fidelity audio, this involves forging connections between the product and the desired auditory experience. Brands often meticulously construct narratives that resonate with their target audience by leveraging principles such as the following:

Reciprocity: Offering listeners a trial period, which makes them more likely to believe in and purchase the product [73].

Authority: Using endorsements from reputed audiophiles or musicians who vouch for the product's quality and the validity of burn-in [68].

Social proof: Highlighting testimonials from satisfied customers who have experienced the positive effects of burn-in [74].

Scarcity: Emphasizing the uniqueness or limited availability of a product can make it more desirable and can intensify beliefs in its special properties, including burn-in effects [75].

Brand Narratives, Prestige, and Auditory Expectations

Every brand aspires to be distinct, and this distinction is often crafted through compelling narratives. Within the audiophile industry, brand prestige plays a significant role. A high-end brand, with a legacy of delivering impeccable auditory experiences, possesses an inherent power to shape perceptions [76].

When such a brand suggests or endorses the concept of burn-in, the weight of its assertion is considerable. Users might be predisposed to expect changes in audio quality over time, purely based on the brand's narrative. This expectation, even if subconscious, can be powerful enough to influence actual auditory experiences.

Moreover, branding is not just about explicit messages. The aesthetics of the product, its packaging, the terminology used in its manual or on its website, and even the brand's history play roles in crafting a narrative that influences perception [77]. If every touchpoint with the brand emphasizes precision, quality, and evolution, the idea of a headphone or speaker "maturing" with time (i.e., burn-in) becomes easier to embrace.

Marketing's Dual Role: Promoter or Challenger of Burn-In

In our quest to understand the societal perspectives on burn-in, it is essential to recognize that marketing does not universally promote the phenomenon. The industry is divided into the following:

Proponents: Some brands actively promote the concept. They might include burn-in instructions, recommend a certain number of hours for optimal performance, or even offer pre-burned-in products for a premium. They harness the power of suggestion, fostering an expectation that the device will indeed sound better with time.

Skeptics: Contrarily, some brands challenge the idea. They might assert that their products deliver optimal performance right out of the box, aligning with some scientific studies that negate mechanical burn-in. This stance is equally potent in shaping perceptions, especially among users who value empirical evidence over anecdotal experiences [78].

The dance between marketing, branding, and individual perceptions is intricate. In the debate surrounding burn-in, where subjective experiences often clash with empirical evidence, the power of suggestion via marketing plays an undeniable role. By crafting narratives, whether endorsing or debunking burn-in, brands wield significant influence in shaping the auditory journey of their consumers.

So to truly grasp the enigma of burn-in, it is essential to see it as more than just an auditory or psychological phenomenon. It is a socio-cultural construct, deeply embedded within the intricate interplay of individual experiences and collective narratives. It is a testament to humanity's continuous endeavor to contextualize experiences within the broader societal fabric.

As we reflect on burn-in, it becomes clear that the discourse is not just about auditory nuances or technical specifications. It is a deeper dive into human behavior and the society we inhabit, showcasing our inherent desire for validation, belonging, and understanding within the vast continuum of collective experiences.

Synthesis of the three dimensions

The narrative of burn-in is not solely rooted in the audible, it is a multidimensional tapestry woven with threads of human physiology, cognitive processes, and societal structures. Each dimension offers its own unique lens to understand this enigma, yet, when intertwined, they form a more holistic and intricate picture.

Physiologically, the human auditory system is a marvel of evolutionary design, with mechanisms for both capturing and interpreting auditory signals. This system's intrinsic adaptive capabilities, paired with the susceptibility of our cochlear hair cells to prolonged sound exposure, might offer some insights into why prolonged listening sessions could yield perceived differences in audio quality.

Yet, sound perception is not solely an anatomical affair. Our psychological makeup, encompassing cognitive biases, emotional states, and past auditory experiences, plays an indispensable role in shaping what we hear and how we interpret it. It is here that phenomena like "brain burn-in" gain relevance, suggesting that our brain, as much as our ears, adapts and recalibrates in response to repeated sound exposures.

Overlaying these two dimensions is the powerful societal fabric, wherein communities, marketing narratives, and cultural nuances shape and reinforce burn-in beliefs. Within audiophile communities, peer validation, influential narratives, and shared experiences amplify individual beliefs, often solidifying the ethos surrounding burn-in.

In understanding burn-in, it is paramount to appreciate the interconnectedness of these three dimensions. Separately, each offers a piece of the puzzle, but together, they provide a comprehensive view, emphasizing the need to approach burn-in not as isolated silos but as a rich, multifaceted phenomenon.

Implications for the audiophile community and medical professionals

The intricate interplay of physiological, psychological, and societal factors shaping the burn-in phenomenon presents profound implications for both the audiophile community and the medical field. For audiophiles, acknowledging these dimensions provides a more informed foundation to discuss and experience burn-in. It shifts the conversation from being purely about equipment and auditory adjustments to encompassing broader cognitive and societal influences. Understanding the subjective nature of this experience, amplified by cognitive biases and community dynamics, can promote healthier discussions, reduce unnecessary expenditure on extended burn-in practices, and guide listeners toward a more personalized audio journey.

From a medical standpoint, these insights are useful for professionals in audiology and neurology. Recognizing the role of cognitive biases and sociocultural influences can aid medical professionals in setting realistic expectations for patients using audio equipment like hearing aids. A patient's belief in the burn-in phenomenon might influence their satisfaction with these devices, and addressing these beliefs can foster better patient outcomes. Understanding the complexities of sound perception is vital for patient education. Equipping patients with knowledge about the factors shaping auditory perception can empower them to make informed choices regarding their auditory health and equipment.

Suggested future research avenues

The myriad complexities underlying the burn-in phenomenon, as delineated in this review, accentuate the dire need for empirical studies to validate, refine, or refute the posited observations and theories. Given the confluence of auditory physiology, cognitive biases, and socio-cultural constructs, experimental designs that employ rigorous methodologies spanning these domains will be instrumental. Additionally, longitudinal studies assessing real-time shifts in perception during prolonged sound exposure, coupled with neuroimaging techniques, could unravel the neural correlates of the burn-in experience.

## Conclusions

Concluding thoughts

The phenomenon of audio burn-in sits at a fascinating crossroads of empirical science and personal experience. While debates continue within the audiophile community, it is evident that our perceptions are shaped not just by equipment mechanics but also by our unique auditory physiology and sociocultural influences. By appreciating these complexities, we can foster a more comprehensive understanding of sound perception and its implications.
